# Crossover from BKT-rough to KPZ-rough surfaces for interface-limited crystal growth/recession

**DOI:** 10.1038/s41598-020-70008-y

**Published:** 2020-08-03

**Authors:** Noriko Akutsu

**Affiliations:** Faculty of Engineering, Osaka Electro-Communication University, Hatsu-cho, Neyagawa, Osaka 572-8530 Japan

**Keywords:** Statistical physics, thermodynamics and nonlinear dynamics, Nonlinear phenomena, Phase transitions and critical phenomena, Materials for optics, Theory and computation

## Abstract

The crossover from a Berezinskii–Kosterlitz–Thouless (BKT) rough surface to a Kardar–Parisi–Zhang (KPZ) rough surface on a vicinal surface is studied using the Monte Carlo method in the non-equilibrium steady state in order to address discrepancies between theoretical results and experiments. The model used is a restricted solid-on-solid model with a discrete Hamiltonian without surface or volume diffusion (interface limited growth/recession). The temperature, driving force for growth, system size, and surface slope dependences of the surface width are calculated for vicinal surfaces tilted between the (001) and (111) surfaces. The surface velocity, kinetic coefficient of the surface, and mean height of the locally merged steps are also calculated. In contrast to the accepted theory for (2 + 1) surfaces, we found that the crossover point from a BKT (logarithmic) rough surface to a KPZ (algebraic) rough surface is different from the kinetic roughening point for the (001) surface. The driving force for crystal growth was found to be a relevant parameter for determining whether the system is in the BKT class or the KPZ class. It was also determined that ad-atoms, ad-holes, islands, and negative-islands block surface fluctuations, which contributes to making a BKT-rough surface.

## Introduction

Surface roughness^1,2^ is important both practically, in the theory of crystal growth, and fundamentally, in the basic theory of interface properties. At equilibrium, the Berezinskii–Kosterlitz–Thouless (BKT)^3,4^ roughening phase transition^5–9^ occurs at the roughening temperature $T_{\mathrm{R}}$ on a two-dimensional (2D) low-Miller-index surface (interface), such as the (001) surface, in 3D. For temperatures $T \ge T_{\mathrm{R}}$, the square of the surface width diverges logarithmically as the linear system size *L* increases to infinity (BKT-rough surface).

For scaling, the concept of the self-affine surface (interface) has been successful and has been widely used^10–13^. The surface width shows a Family–Viscek scaling relation and the surface width *W*(*L*, *t*) can be expressed by the following relation:

1 $$\begin{aligned} W(L,t)\sim L^\alpha f(L^{-z}t), \ z=\alpha /\beta , \end{aligned}$$

where *t* is time and the $\alpha$, $\beta$, and *z* exponents are referred to as the roughness, growth, and dynamic exponents, respectively.

The surface growth equation with a non-linear term under a symmetry principle consideration was first proposed by Kardar, Parisi, and Zhang (Kardar–Parisi–Zhang, KPZ)^14^. For a two-dimensional (2D) surface in 3D, the exponents are obtained numerically as $\alpha =0.3869$, $\beta = 0.2398$, and $z=1.6131$^13,15^ (KPZ-rough surface). The values of the exponents have been observed for directed polymers, as well as other systems in the KPZ universality class.

**Figure 1:**
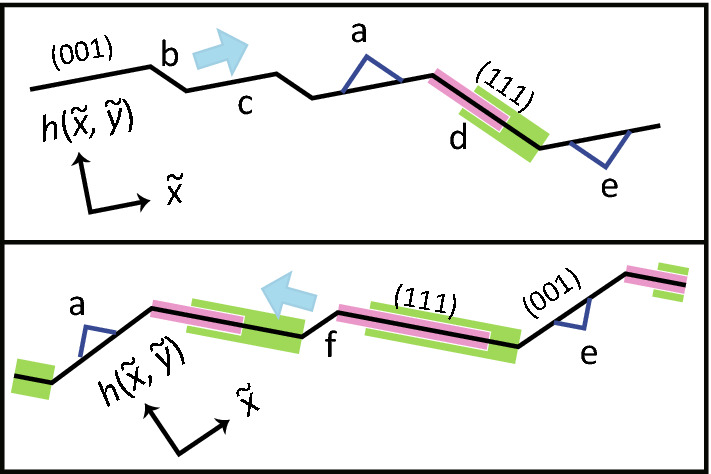
Schematic figures of the side views of vicinal surfaces in the RSOS model. Dark lines: profile of the surfaces. Pink lines: part of the local (111) surface where ad-atoms cannot be added. Green thick lines: part of the local (111) surface where ad-holes cannot form. Upper box: vicinal surface tilted from the (001) surface. Lower box: vicinal surface tilted from the (111) surface. Thick light-blue arrows: step-growth direction for crystal growth. ${\tilde{x}}$ and ${\tilde{y}}$ indicate the $\langle 110 \rangle$ and $\langle {\bar{1}}10 \rangle$ directions. (**a**) ad-atom. (**b**) mono-atomic step. (**c**) (001) terrace. (**d**) step with a height of three mono-atomic steps ; the side surface is (111) surface. (**e**) ad-hole (negative ad-atom). (**f**) negative step.

However, for crystal growth, the experimentally observed exponents are typically different from the KPZ exponents^10,16^. The question of the reason for the difference between KPZ growth and the experimentally observed crystal growth has attracted considerable attention^13,17–21^. For crystal growth with surface diffusion, step-flow growth^1, 17,18^ on a vicinal surface is expected. A vicinal surface at temperatures less than $T_{\mathrm{R}}^{(001)}$ can be described by terrace surfaces and a train of steps, where a step consists of a zig-zag structure on the edge (the terrace, step, kink (TSK) picture^1^, Fig. 1). At equilibrium, the square of the surface width of a vicinal surface diverges logarithmically as the system size diverges^22–25^ for $T<T_{\mathrm{R}}$, similar to a BKT-rough surface. This logarithmic divergence results from long wavelength slope fluctuations^22,23^ caused by step-wandering^9^.

In the non-equilibrium state, one reason why the crystal surface is different from the KPZ class is surface diffusion^10,17,18,26–33^, such as in the case of molecular beam epitaxy (MBE). The surface width shows algebraic divergence but with different values of the exponents from those of KPZ. Depending on the step–step interactions, several groups of exponents are obtained theoretically^20,34^.

Recently, for reaction-limited crystal growth, different exponents were experimentally obtained^35^ from the KPZ values. In addition, in the solution growth of SiC and GaN, self-assembled faceted macrosteps roughen the vicinal surface and degrade the quality of the crystal^36^. In our previous work, we used the restricted solid-on-solid (RSOS) model with a point-contact-type step–step attraction (p-RSOS model) to demonstrate that a faceted macrostep exists stably at equilibrium^37–41^. Here, “restricted” means that the surface height difference between nearest neighbor sites is restricted to $\{0, \pm 1\}$. For interface-limited crystal growth/recession, a faceted macrostep disassembles as the absolute value of the driving force for crystal growth increases^42–45^, which is a different behaviour than that of the results obtained for diffusion limited crystal growth^46^.

Hence, in this article, the crossover from a BKT-rough surface to a KPZ-rough surface for *interface-limited growth* is studied using the Monte Carlo method based on the RSOS model^47–49^ with a discrete Hamiltonian equivalent to the 19-vertex model. The surface is tilted between the (001) surface and the (111) surface. The surface width, surface velocity, and mean height of the locally merged steps are calculated depending on a set of external parameters, temperature *T*, driving force for crystal growth $\Delta \mu$, linear size of the system *L*, and surface slope *p* for the interface limited growth (recession) in the non-equilibrium steady state. The calculated results for the slope dependence of the surface width, surface velocity, and mean height of the locally merged steps are of greatest interest. From these results, we demonstrate which parameter determines whether the surface is KPZ-rough or BKT-rough. This work builds a bridge between mathematical models and surface models for crystal growth.

It should be noted that the RSOS model applied in the present study is slightly different from the RSOS model studied by Kim and Kosterlitz^50^, which corresponds to the absolute SOS (ASOS) or simply the SOS model^51^ for crystal growth, where the height difference between nearest neighbour sites can take a natural number up to the linear system size normal to the surface. However, their numerical simulations based on the KPZ equation were performed for a height difference up to 1. The crossover from a BKT-rough surface to a KPZ-rough surface for a 2D vicinal surface in 3D was first discussed by Wolf^52^ using renormalization calculations with the anisotropic KPZ (AKPZ) equation. However, the present results are different from the AKPZ results on some points.

To obtain clear results for interface-limited crystal growth/recession, the surface diffusion^1,10,17,18,26–33^, volume diffusion^46^, second-nearest-neighbour (2nn) interaction between atoms^53–56^ in crystals, Ehrlich–Schwoebel effect^57,58^, elastic interactions^59^, surface reconstruction^24,25,60^, adsorption effects^61–64^, and point-contact-type step–step attraction^37–41^ are not taken into consideration.

## Model and calculations

The Hamiltonian for a vicinal surface is given by the following equation:

2 $$\begin{aligned} {{{\mathscr {H}}}}= \sum _{\{m,n\}} \left\{ \epsilon [|h(m+1,n)-h(m,n)| \right. + |h(m,n+1)-h(m,n)|] - \Delta \mu \ h(m,n)\} + {{{\mathscr {N}}}} E_{\mathrm{surf}}, \end{aligned}$$

where *h*(*m*, *n*) is the height of the surface at a site (*n*, *m*) , $\epsilon$ is the microscopic ledge energy, ${{{\mathscr {N}}}}$ is the total number of unit cells on the (001) surface, and $E_{\mathrm{surf}}$ is the surface energy per unit cell. The RSOS condition is required implicitly. Here, $\Delta \mu$ is introduced such that $\Delta \mu = \mu _{\mathrm{ambient}} - \mu _{\mathrm{crys}}$, where $\mu _{\mathrm{ambient}}$ and $\mu _{\mathrm{crys}}$ are the bulk chemical potential of the ambient and crystal phases, respectively. At equilibrium, $\Delta \mu =0$; for $\Delta \mu >0$, the crystal grows; whereas for $\Delta \mu <0$, the crystal recedes. The (grand) partition function for the surface at equilibrium is obtained by $Z(T, L, \Delta \mu , N_{\mathrm{step}})|_{\Delta \mu =0} = \sum _{h(m,n)} \exp [ - {{{{\mathscr {H}}}}}/k_{\mathrm{B}}T]$ with a fixed $N_{\mathrm{step}}$.

For first-principles quantum mechanical calculations, $E_{\mathrm{surf}}$ or $\epsilon$ corresponds to the surface free energy, which includes the entropy originating from lattice vibrations and distortions^65^. Hence, $E_{\mathrm{surf}}$ or $\epsilon$ decreases slightly as the temperature increases. However, $E_{\mathrm{surf}}$ and $\epsilon$ are assumed to be constant throughout this work because we concentrate on the crossover phenomena of the surface roughness.

The vicinal surfaces of the tilted (001) and (111) surfaces are considered by using the Monte Carlo method with the Metropolis algorithm. Atoms are captured from the ambient phase to the crystal surface, and escape from the crystal surface to the ambient phase. The number of atoms in a crystal is not conserved. The external parameters are temperature *T*, $\Delta \mu$, number of steps $N_{\mathrm{step}}$, and the linear size of the system *L*. The (mean) surface slope *p* is defined by $p = \tan \theta = N_{\mathrm{step}}a/L$. For details of the Monte Carlo calculations, refer to Ref. [66] and the Supplementary Information.

The square of the surface width *W*(*L*, *t*) is defined by the variance of the height $h(\vec{x},t)$ of the vicinal surface:

3 $$\begin{aligned} gW(L,t)^2&= {} \langle [h(\vec{x},t) - \langle h(\vec{x},t) \rangle ]^2 \rangle , \nonumber \\ g&= {} (1 + p_x^2 + p_y^2) = 1/\cos ^2 \theta , \quad p_x = p_y= N_{\mathrm{step}}a\sqrt{2}/L, \end{aligned}$$

where $\vec{x}$ is a site on the surface, *g* is the determinant of the first fundamental quantity of a curved surface^23, 66^, and $\theta$ is the tilt angle inclined towards the $\langle 111 \rangle$ direction from the $\langle 001 \rangle$ direction.

**Figure 2:**
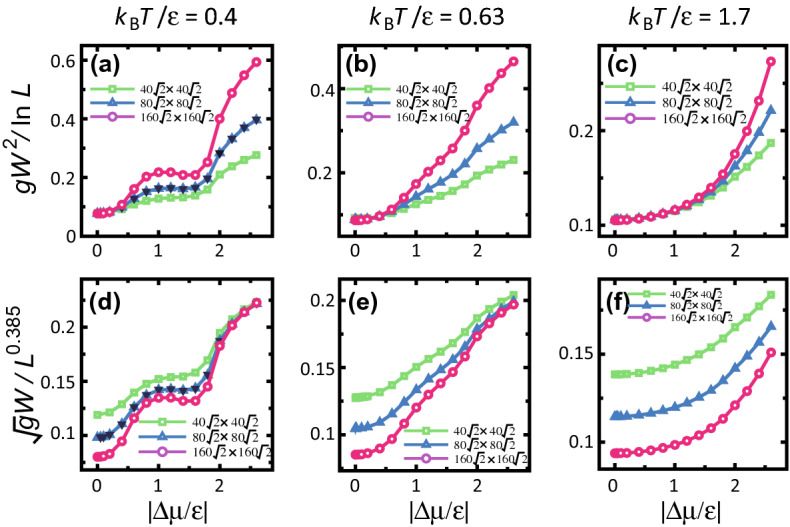
Driving force dependence of the surface width. (**a**)–(**c**) show $gW^2/\ln L$ vs. $\Delta \mu / \epsilon$. (**d**)–(**f**) show $\sqrt{g}W/L^{0.385}$ *vs.* $\Delta \mu / \epsilon$. (**a**), (**c**), (**d**), and (**f**): surface slope $p=3\sqrt{2}/8 \approx 0.530$, tilt angle $\theta$ = 27.9$^\circ$. (**b**, **e**) surface slope $p=\sqrt{2}/2 \approx 0.707$, tilt angle $\theta$ = 35.3$^\circ$. Reverse triangles in (**a**, **d**): $\Delta \mu$ negative and $L=80\sqrt{2}a$ ($a=1$).

**Figure 3:**
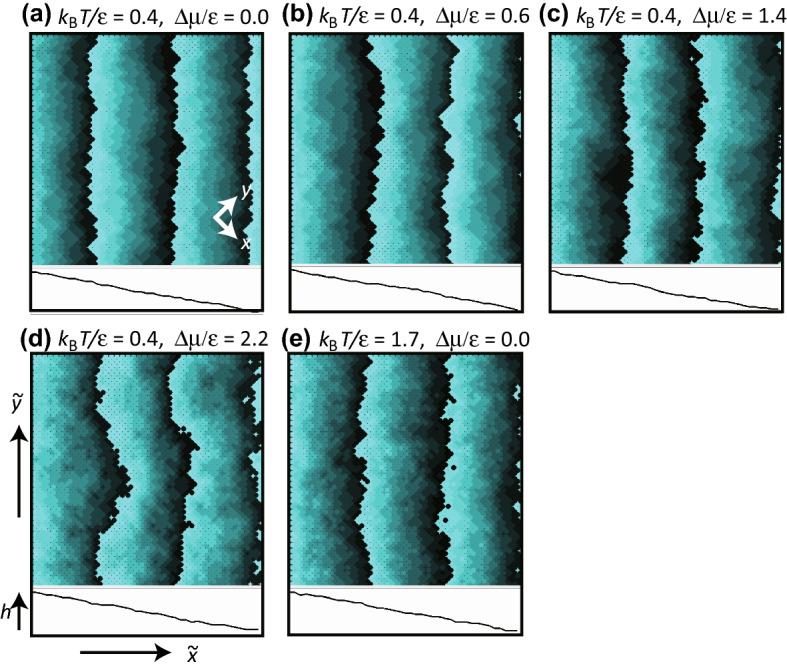
Snapshots of simulated surfaces at $4 \times 10^8$ MCS/site. (**a**,**e**): BKT-rough surfaces. (**b**–**d**): KPZ-rough surfaces. Size: $40\sqrt{2}\times 40\sqrt{2}$. $N_{\mathrm{step}}$ = 30. $p =N_{\mathrm{step}}a/L =3\sqrt{2}/8\approx 0.530$. $\theta =27.9$ degree. The surface height is represented by brightness with 10 gradations, where brighter regions are higher. Due to the finite gradation, where the darkest areas sit next to the brightest areas, the darker area is higher by one gradation unit. The lines showing the side views are drawn with respect to the height along the bottom edge of the top-down views.

## Results

### $\Delta \mu$ and *T* dependence

Figure 2 shows the $|\Delta \mu |$ dependence of the surface width for several temperatures. The roughening temperature of the (001) surface is $T_{\mathrm{R}}^{(001)}/\epsilon = 1.55 \pm 0.02$^38,48^, whereas the roughening temperature of the (111) surface $T_{\mathrm{R}}^{(111)}$ is infinite. The temperature in (c) and (f) is higher than $T_{\mathrm{R}}^{(001)}$.

Near equilibrium ($\Delta \mu \sim 0$), the values of $gW^2/\ln L$ for each system size coincide (Fig. 2a,b,c), whereas for large $|\Delta \mu|$, $\sqrt{g}W/L^\alpha$ with $\alpha =0.385$ for each system size converge as the driving force increases (Fig. 2d,e,f). The value of $\alpha =0.385$ is the KPZ-exponent in this article. Then, a crossover-driving-force $\Delta \mu _{co}$ is introduced. For $|\Delta \mu | < \Delta \mu _{co}$, $W^2 \propto \ln L$ (BKT rough), whereas for $\Delta \mu _{co}< |\Delta \mu |$, $W \propto L^\alpha$ (algebraic rough). From Fig. 2, it is clear that the value of $\Delta \mu _{co}$ depends on temperature. For high $|\Delta \mu|$, the convergence of $\alpha$ to the KPZ value is stronger when the temperature is lower. At $k_{\mathrm{B}}T/\epsilon =0.4$, $\Delta \mu _{co}|_{k_{\mathrm{B}}T/\epsilon =0.4} = 0.3 \epsilon$ (Fig. 2a). For $|\Delta \mu /\epsilon |>2$, there is good agreement between the three lines for $\sqrt{g}W/L^{0.385}$ (Fig. 2d).

It has been suggested that a KPZ-rough surface may appear when the surface is kinetically roughened^10,18,52^ because islands on the terraces enhance the step-growth velocity. Hence, the driving force for the kinetic roughening $\Delta \mu _{kr}^{(001)}$ on the (001) surface is studied. The obtained $\Delta \mu _{kr}^{(001)}$ at $k_{\mathrm{B}}T/\epsilon =0.4$ is $\Delta \mu _{kr}^{(001)}/\epsilon =1.15 \pm 0.15$. $\Delta \mu _{kr}^{(001)}$ is determined as follows. For a smooth terrace surface, the surface velocity *V* on the (001) surface converges to zero as the surface slope $p \rightarrow 0$; whereas, for a rough terrace surface, *V* on the (001) surface converges to a finite value as $p \rightarrow 0$. Then, $\Delta \mu _{kr}^{(001)}$ is determined as the largest $|\Delta \mu |$, so that the surface velocity *V* converges to zero as the slope $p \rightarrow 0$^52^ (refer to the section on the surface velocity below). In Fig. 2a,d, *W* near $\Delta \mu _{kr}^{(001)}$ (around $\Delta \mu /\epsilon =1$) seems to form a broad peak for larger system sizes. This peak in *W* is considered to relate to the kinetic roughening of the (001) surface. In snapshots of the surface (Fig. 3), the steps rarely have an overhang structure (Fig. 3a,b) for $\Delta \mu <\Delta \mu _{kr}$, whereas overhang structures on the contour lines can be seen for Fig. 3c,d. Figure 3e shows the thermally roughened surface.

It is interesting that the kinetic roughening occurs approximately where the linear size of the 2D critical nucleus on a (001) terrace is less than 2*a* ($a=1$), where *a* is the lattice constant. Assuming that the shape of the critical nucleus on a (001) terrace is square, the size of the critical nucleus $r^*$ is expressed by $r^*/a=2\epsilon /\Delta \mu$. At $|\Delta \mu /\epsilon |=1$ or 2, $r^*/a=2$ or 1, respectively. Islands with a compact shape are frequently formed near $|\Delta \mu /\epsilon | = 1.0$ and merge with the step on the same layer. The step edge consists of several 1D “overhang” structures due to merging of the islands with steps. In this manner, islands on a terrace enhance the step velocity. For $r^*/a \le 1$, where $\Delta \mu /\epsilon \ge 2$, even a single atom on the terrace grows to form an island. This relates to the fact that *W* increases drastically around $\Delta \mu /\epsilon = 2$.

It should be noted that the TSK picture is broken for $|\Delta \mu | \ge \Delta \mu _{kr}$ or for $T>T_{\mathrm{R}}$, since the “step” is not well-defined due to the terrace being roughened. However, the contour lines on the surface shown in Fig. 3c,d,e (and Supplementary Fig. S1 (b), (c), and (d)) show the complexities of the surface. In Fig. 3d and Supplementary Fig. S1 (c), dendritic contour shapes can be seen.

In the case of crystal recession, the 2D nucleus on the (001) terrace at $\Delta \mu /\epsilon = -1$ is a *negative* square nucleus. Here, an ad-hole, a negative-island, and a negative-nucleus are, respectively, a vacancy on the terrace, an island made by a vacancy, and a negative-island with a critical size.

At $k_{\mathrm{B}}T/\epsilon = 0.63$, the characteristics of the $|\Delta \mu |$ dependence of *W* are similar to those at $k_{\mathrm{B}}T/\epsilon = 0.4$. We also have $\Delta \mu _{co}|_{k_{\mathrm{B}}T/\epsilon =0.63} = 0.5 \epsilon$, whereas $\Delta \mu _{kr}|_{k_{\mathrm{B}}T/\epsilon =0.63} = 0.65\epsilon \pm 0.05\epsilon$. The value of *W* is smaller than that for $k_{\mathrm{B}}T/\epsilon = 0.4$; the peak of *W* around $\Delta \mu _{kr}|_{k_{\mathrm{B}}T/\epsilon =0.63}$ is small.

At $k_{\mathrm{B}}T/\epsilon =1.7$ where $T>T_{\mathrm{R}}^{(001)}$, the (001) terraces are rough at $\Delta \mu =0$. Hence, there is no kinetic roughening. Here, $\Delta \mu _{co}|_{k_{\mathrm{B}}T/\epsilon =1.7} = 1.2 \epsilon$, which is the largest among the three cases. Multi-layer island formation caused by thermal fluctuations increases the region of BKT roughening (Fig. 3e).

**Figure 4:**
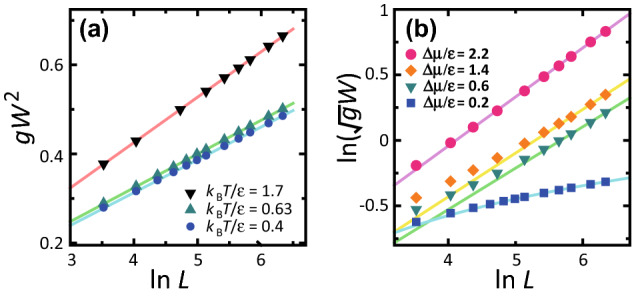
System-size dependence of surface width. $p=3\sqrt{2}/8 \approx 0.530$. (**a**) $gW^2$ *vs.* $\ln L$ at $\Delta \mu = 0$. Lines: from the top, $gW^2=0.0174+0.102 \ln L$, $gW^2=0.0201+0.0759 \ln L$, and $gW^2=0.0178+0.0738 \ln L$. (**b**) $\ln (\sqrt{g}W)$ vs. $\ln L$. $k_{\mathrm{B}}T/\epsilon =0.4$. Lines: from the top, $\sqrt{g}W=0.214 L^{0.374}$, $\sqrt{g}W=0.175 L^{0.331}$, $\sqrt{g}W=0.167 L^{0.316}$, and $gW^2=-0.0497+0.0917 \ln L$.

### *L* dependence

Figure 4 shows the $\ln L$ dependence of $gW^2$ and $\ln (\sqrt{g}W)$. Figure 4a shows the results at equilibrium. In contrast to the two-component system of our previous work at equilibrium^67^, the linearity of the obtained data is high. This indicates that the elementary steps are well separated and the intervals between kinks are small relative to the system size. The amplitudes of the lines at $p= 3\sqrt{2}/8$ increase as the temperature increases, specifically, 0.102, 0.0759, and 0.0738 for $k_{\mathrm{B}}T/\epsilon =1.7$, 0.63, and 0.4, respectively. These amplitudes are larger than the universal value of $1/(2\pi ^2) \approx 0.0507$ for $p \rightarrow 0$^24,68,69^.

Figure 4b shows results for the non-equilibrium steady-state at $k_{\mathrm{B}}T/\epsilon =0.4$. For small $\Delta \mu$, $gW^2$ increases logarithmically as the system size increases. However, a power law behaviour of $\sqrt{g}W$ is obtained for relatively large $|\Delta \mu |$. The slopes of the lines show the roughness exponent $\alpha$. For large *L*, the obtained $\alpha$ for $\Delta \mu /\epsilon =2.2$, 1.4, and 0.6 are 0.347, 0.331, and 0.316, respectively. This is consistent with the results seen in Fig. 2d–f. The exponent $\alpha$ seems to gradually increase as $|\Delta \mu |$ increases. However, the slope at larger *L* is steeper. Therefore, we consider that the exponent $\alpha$ converges to the KPZ value in the limit of $L \rightarrow \infty$. The large finite size effect decreases the value of $\alpha$ in the small length region.

It is interesting that large wavelength surface fluctuations are observed in the snapshots in Fig. 3b,c,d. We also show snapshots for $L=400\sqrt{2}a$ in the Supplementary Information.

From the results in this and the previous sections, we conclude that the crossover point $\Delta \mu _{co}$ between the BKT-rough and the algebraic-rough surfaces is different from the kinetic roughening point $\Delta \mu _{kr}$. Also, the algebraic-rough surface is essentially the KPZ-rough surface in the limit of $L \rightarrow \infty$. The large finite size effect decreases the value of $\alpha$ for the small system size.

**Figure 5:**
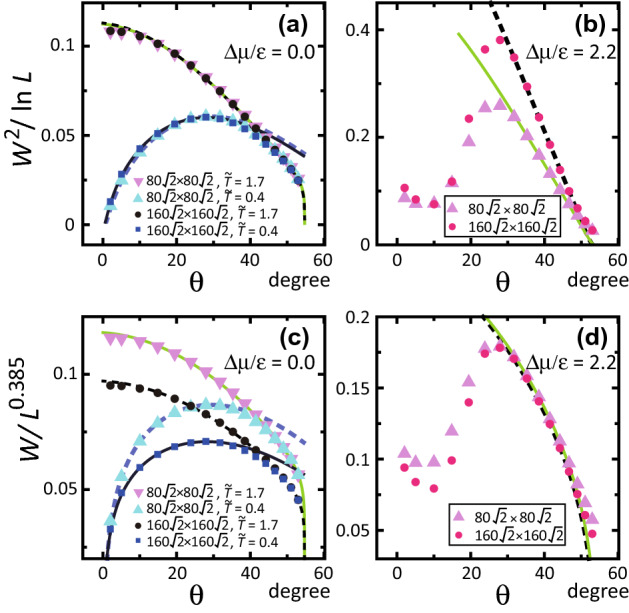
Slope dependence of *W*. $p=\tan \theta$. $g= 1+p^2$. ${\tilde{T}}=k_{\mathrm{B}}T/\epsilon$. (**a**, **c**) Dark solid line: $\sqrt{g}W=(0.317+ 0.0622 \ln p)\sqrt{\ln L}$. Light broken line: $\sqrt{g}W=(0.321+ 0.0677 \ln p)\sqrt{\ln L}$. Light solid line: $\sqrt{g}W=[0.327+ 0.0249 \ln (\sqrt{2}-p)]\sqrt{\ln L}$. Dark broken line: $\sqrt{g}W=[0.327+ 0.0269 \ln (\sqrt{2}-p)]\sqrt{\ln L}$. (**b**, **d**) $k_{\mathrm{B}}T/\epsilon = 0.4$. Light solid line: $\sqrt{g}W=[0.233+ 0.0836 \ln (\sqrt{2}-p)]L^{0.374}$. Dark broken line: $\sqrt{g}W=[0.233+ 0.0886 \ln (\sqrt{2}-p))]L^{0.374}$.

### *p* dependence

Figure 5 shows the slope dependence of *W* and $W^2$. At equilibrium, apart from the neighbourhood of the (001) surface, *W* for $k_{\mathrm{B}}T/\epsilon =0.4$ is well described by (Fig. 5a) the following single equation:

4 $$\begin{aligned} gW^2/(\ln L) = (A + B \ln p)^2, \quad A=0.319\pm 0.006, \ B= 0.065 \pm 0.008. \end{aligned}$$

Here, for $T> T_{\mathrm{R}}^{(001)}$, $gW^2/\ln L$ of the (001) surface converges to a finite value for $p \rightarrow 0$. For different temperatures for $T<T_{\mathrm{R}}^{(001)}$, the slope dependence of *W* agrees well within 5%.

For a large surface slope, near the (111) surface where $T_{\mathrm{R}}^{(111)}$ is infinite, *W* is well described by the following single equation:

5 $$\begin{aligned} gW^2/(\ln L) = [A' + B' \ln (\sqrt{2}-p)]^2, \quad A'= 0.327 \pm 0.002, \ B' = 0.026 \pm 0.005, \end{aligned}$$

for $k_{\mathrm{B}}T/\epsilon =$ 0.4, 0.63, and 1.7. It should be noted that the vicinal surface around the (111) surface of the RSOS model is approximate compared to the real (111) surface. The negative-step (Fig. 1) is a “step” with a (111) terrace in the step-down direction. However, due to the geometrical restrictions of the model, there are no “steps” with a (111) terrace in the step-up direction. For the same reason, there are no ad-atoms or ad-holes on the (111) terraces either.

For the non-equilibrium steady state, from Fig. 5d, apart from the neighbourhood of the (111) surface, the slope dependence of *W* is well described by

6 $$\begin{aligned} gW/L^{\alpha }=A'' + B'' \ln (\sqrt{2}-p), \quad A''= 0.233 \pm 0.002, \ B''= 0.086 \pm 0.004, \quad \alpha =0.374, \end{aligned}$$

at $$|\Delta \mu /\epsilon |= 2.2$$ and $$k_{\mathrm{B}}T/\epsilon =0.4$$.

Unexpectedly, as seen from Fig. 5b,d for small $\theta$, a vicinal surface with a small tilt angle shows a different behaviour from the KPZ-rough surface even if $|\Delta \mu |$ is high. For $\theta < 19^\circ$, the vicinal surface is BKT-rough. We will return to this point in the discussion.

**Figure 6:**
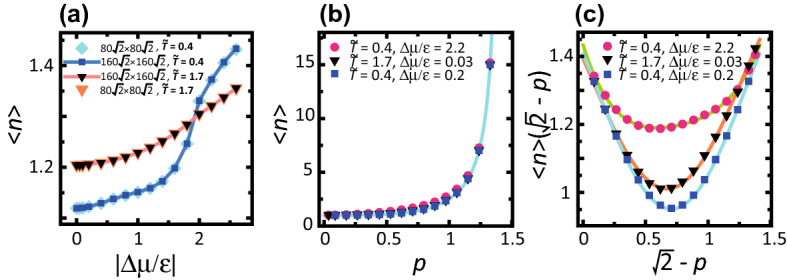
$|\Delta \mu |$ and the slope dependence of the mean height of a locally merged step $\langle n \rangle$. ${\tilde{T}}=k_{\mathrm{B}}T/\epsilon$. (**a**) $|\Delta \mu |$ dependence of $\langle n \rangle$. $p=3\sqrt{2}/8 \approx 0.530$. $\theta = 27.9^\circ$. (**b**) Slope dependence of $\langle n \rangle$. (**c**) Slope dependence of $\langle n \rangle (\sqrt{2}-p)$. Lines: $\langle n \rangle (\sqrt{2}-p) = A+B(\sqrt{2}-p-C)^2+D(\sqrt{2}-p-C)^3+E(\sqrt{2}-p-C)^4$; from top to bottom, $A=1.19$, $B= 0.290$, $C=0.594$, $D=-0.352$, and $E=0.535$; $A=1.01$, $B= 1.07$, $C=0.672$, $D=0$, and $E=-0.493$; and $A=0.951$, $B= 1.21$, $C=0.707$, $D=0$, and $E=-0.604$.

### Mean height of locally merged step

Figure 6a shows the $\Delta \mu$ dependence of the mean height of locally merged steps $\langle n \rangle$. In contrast to the cases of surfaces with faceted macrosteps^43–45^, $\langle n \rangle$ is independent of the system size or the initial configurations. This lack of a finite size effect means that $\langle n \rangle$ in the RSOS model is determined by the local or short wavelength structure of steps.

It is interesting that $\langle n \rangle$ at $k_{\mathrm{B}}T/\epsilon =0.4$ increases rapidly for $\Delta \mu /\epsilon >1.2$, which is almost the same as $\Delta \mu _{kr}$ at $k_{\mathrm{B}}T/\epsilon =0.4$. At equilibrium, the result that $\langle n \rangle \sim 1$ indicates that the steps are well separated. When $|\Delta \mu |$ is about $\Delta \mu _{kr}$, 2D nucleation with a compact shape and growth occurs frequently on the (001) terraces (Fig. 3c, Supplementary Fig. S1 (b)). The growing islands merge with the step on the same layer to enhance the surface growth velocity. However, growing islands that catch up with steps on the lower layer are prevented from further growth due to geometrical restrictions. Hence, the ratio of multi-height steps increases. This is why $\langle n \rangle$ increases rapidly as $|\Delta \mu |$ increases for $|\Delta \mu |> \Delta \mu _{kr}$. Assuming that the increase of $\langle n \rangle$ is dominantly caused by the formation of double steps, the ratio of the double step is less than 20% for $\Delta \mu /\epsilon \le 1.6$, whereas the ratio increases up to about 50% for $\Delta \mu /\epsilon > 1.8$ as $\Delta \mu$ increases.

Again, it should be noted that when $|\Delta \mu |$ exceeds $\Delta \mu _{kr}$, the TSK picture breaks down. However, regarding a contour line on the surface as an extended meaning of a “step”, the complexity of the surface undulations can be explained by an extended T“S”K picture.

At high $|\Delta \mu |>2$, since the size of the critical nucleus is less than one, ad-atoms on the terrace frequently grow larger islands for crystal growth. Also, the ad-atoms rarely escape from the terrace and the islands have dendrite shapes. Hence, by merging to a step, the contour lines of the vicinal surface exhibit winding shapes (Fig. 3d, Supplementary Fig. S1 (c)).

The slope dependence of $\langle n \rangle$ was also calculated (Fig. 6b). $\langle n \rangle$ is approximated by $\langle n \rangle \approx A\sqrt{2}/(\sqrt{2}-p)$ with $A=0.672$ for $k_{\mathrm{B}}T/\epsilon =0.4$ and $\Delta \mu /\epsilon = 0.2$. More precisely, $\langle n \rangle$ is relatively large near the (001) and (111) surfaces (Fig. 6c). For a BKT-rough surface, $\langle n \rangle (\sqrt{2}-p)$ is well expressed by a quadratic function with respect to $(\sqrt{2}-p)$; whereas for a KPZ-rough surface, $\langle n \rangle (\sqrt{2}-p)$ is asymmetric around $p=1/\sqrt{2}$.

**Figure 7:**
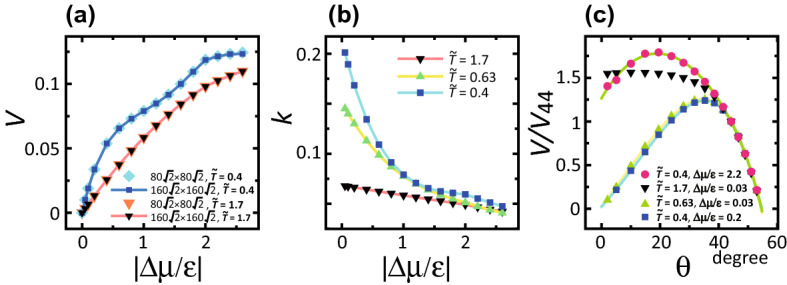
**a** Surface growth velocity *V*. $p=3\sqrt{2}/8 \approx 0.530$. $\theta = 27.9^\circ$. ${\tilde{T}}=k_{\mathrm{B}}T/\epsilon$. **b** Kinetic coefficient $k = V \epsilon \tau /(a\Delta \mu )$. $p=3\sqrt{2}/8 \approx 0.530$ for ${\tilde{T}}=1.7$ and ${\tilde{T}}=0.4$. $p=\sqrt{2}/2 \approx 0.707$ for ${\tilde{T}}=0.63$. $\theta = 35.3^\circ$. **c** Slope dependence of the relative surface velocity. $V_{44}$ is the surface velocity at $\theta =44^{\circ }$. Lines: $V/V_{44} = A+B(p-C)^2 +D(p-C)^3 +E(p-C)^4$, $p=\tan \theta$; from top to bottom, $A=1.78$, $B= -3.26$, $C=0.345$, $D=2.68$, and $E= -1.03$; $A=1.25$, $B= -3.26$, $C=0.694$, $D=0$, and $E= 1.46$; $A=1.24$, $B= -3.45$, $C=0.704$, $D=0$, and $E= 2.01$.

### Surface velocity

Figure 7a shows the $\Delta \mu$ dependences of the surface velocity *V*. The surface velocity does not depend on the system size, but is determined by the local structure of the surface, such as the kink density on the surface. To determine the $|\Delta \mu |$ dependence of the kink density, the kinetic coefficient $k= (V/\Delta \mu )(\epsilon \tau /a)$^46^ was calculated, where $\tau$ is the interval time of one MCS/site (Fig. 7b). Unexpectedly, for $T<T_{\mathrm{R}}$, the kinetic coefficient *k* decreases rapidly as $|\Delta \mu |$ increases up to $\Delta \mu _{kr}$; *k* decreases gradually for $|\Delta \mu |>\Delta \mu _{kr}$. The change of *k* happens in the step flow region rather than in the kinetically roughened region. For $T> T_{\mathrm{R}}^{(001)}$, *k* decreases by a constant rate as $|\Delta \mu |$ increases.

Considering the surface velocity, a stepwise increase can be seen in Fig. 7a for $k_{\mathrm{B}}T/\epsilon = 0.4$. The step flow growth is almost saturated around $k_{\mathrm{B}}T/\epsilon \sim 1$. For $|\Delta \mu /\epsilon | > \Delta \mu _{kr}$, regarding the contour lines as extended surface “steps”, additional surface growth (or recession for $\Delta \mu <0$) occurs by a 2D dendritic-island-growth process. Hence, the surface growth shows a stepwise increase. A stepwise increase of *V* with respect to $\Delta \mu$ is also observed experimentally for a metal-alloy surface^70^. It is known that the islands on the terrace surface have dendritic shapes, which is consistent with the present observation of the contour shapes in the computer simulations (Fig. 3d, Supplementary Fig. S2 (c)).

Figure 7c shows the slope dependence of the relative surface velocity. $V_{44}$ is the surface velocity of the surface with $\theta = 44^{\circ }$. $V/V_{44}$ is equal to $k/k_{44}$, where $k_{44}$ is the kinetic coefficient of the surface with $\theta = 44^{\circ }$. In the limit of $\theta =0$, $V/V_{44}$ for a thermally rough (001) surface and a kinetically rough (001) surface converge to finite values; whereas for a smooth (001) surface, $V/V_{44}$ converges to zero. Using these characteristics, $\Delta \mu _{kr}$ can be determined.

It is remarkable that, for $\theta > 42^{\circ }$ or $p> 0.90$, the slope dependence of $V/V_{44}$ coincides well with one of the curves, regardless of the difference of *T* or $\Delta \mu$. This indicates that the local structure or the short range structure of the vicinal surface for $\theta > 42^{\circ }$ is approximately the same.

## Discussion

The relationship between the surface velocity and fluctuation width was discussed by Wolf^52^ using the renormalization group method. Let us consider the AKPZ model^17,52^:

7 $$\begin{aligned} \frac{\partial h}{\partial t}&= {} \nu _{{\tilde{x}}} \frac{\partial ^2 h}{\partial {\tilde{x}}^2} + \nu _{{\tilde{y}}} \frac{\partial ^2 h}{\partial {\tilde{y}}^2} + \frac{\lambda _{{\tilde{x}}}}{2} \left( \frac{\partial h}{\partial {\tilde{x}}}\right) ^2 +\frac{\lambda _{{\tilde{y}}}}{2} \left( \frac{\partial h}{\partial {\tilde{y}}}\right) ^2+ \eta (\vec{x},t), \nonumber \\&\langle \eta (\vec{x},t) \rangle = 0, \quad \langle \eta (\vec{x},t)\eta (\vec{x}',t') \rangle = D\delta (\vec{x}-\vec{x}')\delta (t-t'), \end{aligned}$$

where $\nu _{{\tilde{x}}}$ and $\nu _{{\tilde{y}}}$ are relaxation constants for the ${\tilde{x}}$ and ${\tilde{y}}$ directions, respectively, related to the surface tension, $\lambda _{{\tilde{x}}}$ and $\lambda _{{\tilde{y}}}$ are the coefficients related to the “excess velocity”, and $\eta (\vec{x},t)$ is Gaussian white noise. The parameters $\lambda _{{\tilde{x}}}$ and $\lambda _{{\tilde{y}}}$ are given by

8 $$\begin{aligned} \lambda _{{\tilde{x}}}=\partial ^2 V/\partial p^2, \ \lambda _{{\tilde{y}}}=(\partial V/\partial p)/p. \end{aligned}$$

Wolf^52^ found that for $\lambda _{{\tilde{x}}} \lambda _{{\tilde{y}}} > 0$, the system converges to a fixed point with algebraic roughness ($W \propto L^\alpha$), whereas for $\lambda _{{\tilde{x}}}\lambda _{{\tilde{y}}}<0$, the system converges to another fixed point with logarithmic roughness ($W^2 \propto \ln L$).

To compare our results to the AKPZ model, we investigate the consistency between our results and the AKPZ results. For small $\theta$, $\partial V/\partial p >0$ and $\partial ^2V/\partial p^2<0$. Then, $\lambda _{{\tilde{x}}}\lambda _{{\tilde{y}}}<0$ indicates that the surface should be BKT (logarithmic)-rough, which is consistent with our results. For large $\theta$, $\partial V/\partial p <0$ and $\partial ^2V/\partial p^2<0$. Then, $\lambda _{{\tilde{x}}}\lambda _{{\tilde{y}}}>0$ indicates that the surface is KPZ (algebraic)-rough. This seems to be consistent with the results for the large $|\Delta \mu |$ case. However, for small $|\Delta \mu |$, our results show BKT roughness for large $\theta$. Hence, the AKPZ results are not fully consistent with our results. More seriously, if the surface slope *p* is replaced by $\sqrt{2}-p$ and redefined by ${\hat{p}}$, then we have $\partial V/\partial {\hat{p}} >0$ and $\partial ^2V/\partial {\hat{p}}^2<0$ for large $\theta$ surfaces, which leads to logarithmic roughness. The AKPZ results change depending on the definition of the slope *p*. Therefore, the AKPZ results cannot be established for the case of large $\theta$ in our model.

Then, the question remains as to what is the “relevant” quantity to obtain a KPZ (algebraic)-rough surface from a BKT-rough surface. We focused on the difference between the surfaces of $k_{\mathrm{B}}T/\epsilon =0.4$ with $|\Delta \mu |=0$ and $|\Delta \mu /\epsilon |=2.2$ for $\theta > 30^\circ$. The only difference between these surfaces in the external parameters is the value of $|\Delta \mu |$. Therefore, we conclude that a sufficiently large $|\Delta \mu |>0$ creates a KPZ (algebraic)-rough surface.

The next question is why a large $|\Delta \mu |>0$ creates a KPZ (algebraic)-rough surface. We consider that a sufficiently strong asymmetry between the attachment and detachment of atoms, which creates overhang structures on negative-step^40^ edges, gives rise to the KPZ-rough surface. Here, a negative-step is a step such that the terrace is a (111) surface and the side surface of the negative-step is a (001) surface (Fig. 1). For large $\theta$, where the vicinal surface is close to the (111) surface, the surface grows or recedes at the edge of negative-steps on the (111) surface (Supplementary Fig. S2). Due to the geometric restrictions of the RSOS model, ad-atoms or ad-holes are forbidden on the (111) surface. Hence, the (111) surface does not roughen kinetically. Nevertheless, the surface becomes a KPZ-rough surface when $|\Delta \mu |$ is sufficiently large. Therefore, we conclude that a sufficiently strong asymmetry between attachment and detachment of atoms at negative-step edges creates the KPZ-rough surface.

Physically, for $|\Delta \mu /\epsilon |>>0$ with large $\theta$, growing negative-steps have a strongly anisotropic step velocity. To describe this anisotropy, we introduce Miller indices for the (111) plane. The (01), (10), and (11) negative-steps have 2D vectors normal to the mean step-running directions in the $\langle {\bar{1}}01 \rangle$, $\langle 0{\bar{1}}1 \rangle$, and $\langle 1{\bar{1}}0 \rangle$ directions, respectively. Since the step velocity of (11) negative-steps is larger than that for (01) or (10) negative-steps, (11) negative-steps with a small-scale zig-zag structure involving (01) and (10) negative-steps under non-equilibrium conditions tend to be surrounded by longer (01) and (10) steps. Then, larger square shapes with (01) and (10) negative-steps are formed in non-equilibrium conditions than at equilibrium (Supplementary Fig. S2). Some produce an overhanging structure at the negative-step edges. In this manner, a large-scale zig-zag structure with overhangs on the negative-step edges is formed due to the anisotropy in the step velocity for $|\Delta \mu /\epsilon |>>0$, which increases the width of surface fluctuations. Therefore, the anisotropy in the step velocity or the kink density at the step edges creates KPZ-roughness on the surface for $|\Delta \mu /\epsilon |>>0$.

The third question is why a vicinal surface with small $\theta$ shows a BKT-rough surface even for large $|\Delta \mu |$. We consider that ad-atoms, ad-holes, islands, and negative-islands on “terraces” block the advancement/recession of the “steps”, decreasing the surface fluctuation width. The only difference between a BKT-rough surface and a KPZ-rough surface is the surface slope for $k_{\mathrm{B}}T/\epsilon =0.4$ and $\Delta \mu /\epsilon = 2.2$. For a small $\theta$ surface, ad-atoms, ad-holes, islands, and negative-islands form on the (001) terrace (Figs. 1, Fig. 3b–d). When these excitations exist on the same layer as a step, they help to grow/recede the step. However, when such excitations exist on different layers from the step, they hinder the advancement/recession of the step. In this manner, the surface fluctuation is suppressed, decreasing *W*. For a large $\theta$ surface, ad-atoms, ad-holes, islands, and negative-islands cannot form on the (111) terrace due to geometrical restrictions. The surface can grow/recede mainly by growing/receding steps and negative-steps. A similar situation occurs for a 2D lattice gas on a surface. The phase transition in the 2D lattice gas model belongs to the 2D Ising class. However, due to the presence of islands with multiple heights on the surface, the roughening transition of the surface belongs to the BKT class. Therefore, we conclude that the ad-atoms, ad-holes, islands, and negative-islands are relevant to making the BKT-rough surface.

In a real surface, there exist many other elements, as mentioned at the end of the introduction. Since the atomic attachment/detachment process on the vicinal surface studied in the present work is fundamental, examining the combinations with other elements should provide new knowledge. However, detail studies of the combinations are left as future work. In the following, we will discuss the combination with surface diffusion and the combination with the 2nd nn interactions between atoms.

Surface diffusion is one of the most important processes for crystal growth^1^. Hence, extensive computer simulation studies of surface diffusion have been undertaken^26–33,71,72^. Since the diffusion process of atoms is a result of the mass conservation law of atoms, the universality class of a surface with a diffusion process must be different from the universality class of a surface without diffusion^73^. When surface diffusion occurs along with an atomic attachment/detachment process, it is referred to as a “desorption” process in statistical mechanics. The competition between the diffusion process and the desorption process causes a crossover phenomenon on the surface between a diffusion-relevant-rough surface and a desorption-relevant-rough surface^10,26–33^ for a kinetically roughened surface. Here, the roughness exponent $\alpha$ for the diffusion-relevant-rough surface is known to be $\alpha =(4-D)/3 \approx 0.667$ with $D=2$ for a 2D surface in 3D^28^.

The occurrence of the crossover between the diffusion-relevant-rough surface and desorption-relevant-rough surface is system-size dependent, as measured by the surface width, with the diffusion length $\lambda _s$ being the transition boundary: for $L<\lambda _s$, the diffusion process is relevant, while for $\lambda _s < L$, the desorption process is relevant^10^. Here, the diffusion length $\lambda _s$, which is a characteristic length for the surface diffusion, is expressed by^1^

9 $$\begin{aligned} \lambda _s^2 = D_s \tau _s, \quad D_s = a^2/\tau _h, \quad 1/\tau _s = \nu \exp (-W'_s/k_{\mathrm{B}}T), \quad 1/\tau _h = \nu ' \exp (-U_h/k_{\mathrm{B}}T), \end{aligned}$$

where $D_s$ is the diffusion coefficient for an atom travelling on the surface, $\tau _s$ is the mean life of an adsorbed atom before being evaporated, $\tau _h$ is the mean hopping time for an atom, $W'_s$ is the evaporation energy of an atom, $U_h$ is the activation energy between two neighbouring equilibrium positions on the surface, and $\nu$ and $\nu '$ are frequency factors. For many semiconductors, $U_h<< W'_s$, for which $\tau _s$ is large. Hence, the number of atoms on the surface is approximately conserved for a finite area $L^2$. Hence, the diffusion length is determined by $D_s$ and increases as the temperature increases. This is the situation often observed in molecular beam epitaxy (MBE). While for metals, $U_h < W'_s$, and $\tau _s$ determines the diffusion length. Hence, the diffusion length becomes shorter as the temperature increases. Atoms detach to the ambient phase frequently at high temperatures^1^.

It should be noted that based on the present study, the desorption-relevant-rough surface for $\lambda _s<L$ can be a BKT-rough surface, though the surface has been thought to be KPZ-rough. For a vicinal surface, a terrace with a (001) surface is smooth for small $|\Delta \mu |$ less than the kinetic roughening point $\Delta \mu _{kr}$ at temperatures less than the roughening transition temperature of the (001) surface $T_{\mathrm{R}}^{(001)}$. A crossover between the desorption-relevant-rough surface and the diffusion-relevant-rough surface with respect to the surface slope *p* must occur even if the (001) surface is smooth. When the mean terrace width $\ell =a/p$ is less than $\lambda _s$, the vicinal surface should be a 1D diffusion-relevant-rough surface^17,26^, even though $\lambda _s<L$. Thus, the slope dependence of the surface width of a vicinal surface where diffusion and desorption processes coexist will be complex. The crossover between the desorption-relevant-rough surface and the diffusion-relevant-rough surface is intricately intertwined with the crossover between the BKT-rough surface and the KPZ-rough surface. The detail study of these crossovers is a future problem.

The 2nn interactions between atoms^53–56^ change the morphology and anisotropy of the surface (step) tension. Here, a vicinal surface is rough even if the terrace surface is smooth^22–25^. For repulsive 2nn interactions, the 2nn interactions are expected to be “irrelevant” for determining whether the vicinal surface is BKT-rough or KPZ-rough, because the 2nn interaction does not change the Gruber–Mullins–Pokrovsky–Talapov^74,75^ universality class or the 1D free fermion character of mono-atomic steps^68,69,76^ on a vicinal surface at equilibrium. However, considering the pre-roughening^77^ proposed by den Nijs and Rommels on a (001) surface for an SOS model with 2nn interactions between atoms, a new class for the kinetic roughening might exist. A detail study on this is also left as future work.

For an attractive interaction between 2nn atoms, the phenomena can be changed drastically. The RSOS model with point-contact-type step–step attraction (p-RSOS model) partially accounts for the 2nn attractions between atoms. In the p-RSOS model, mono-atomic steps self-assemble to form faceted macrosteps at low temperatures at equilibrium^37–45^. In that case, the surface width is different from the results near equilibrium reported in the present paper. The author is currently preparing a separate article for publication to report these results.

## Conclusions

For the RSOS model with a discrete Hamiltonian under a non-equilibrium steady state without surface diffusion or volume diffusion:

- The crossover point $\Delta \mu _{co}$ between the BKT (logarithmic)-rough surface and the KPZ (algebraic)-rough surface is different from the kinetic roughening point $\Delta \mu _{kr}$.
- A step flow growth or recession leads intrinsically to a KPZ-rough surface due to the anisotropic step velocity, where the anisotropy is caused by the crystal structure.
- The ad-atoms, ad-holes, and their clusters on terraces, which block the step advancement and recession, are relevant for making the BKT-rough surface.

## Supplementary information

Supplementary information.

## References

[1] Burton WK, Cabrera N, Frank FC (1951). The growth of crystals and the equilibrium structure of their surfaces. Philos. Trans. R. Soc. Lond. A.

[2] Weeks JD, Gilmer GH, Leamy HJ (1973). Structural transition in the ising-model interface. Phys. Rev. Lett..

[3] Berezinskii VL (1971). Destruction of long-range order in one-dimensional and two-dimensional systems having a continuous Symmetry Group I. Classical Systems. Sov. Phys. JETP.

[4] Kosterlitz JM, Thouless DJ (1973). Ordering, metastability and phase transitions in two-dimensional systems. J. Phys. C.

[5] Chui ST, Weeks JD (1976). Phase transition in the two-dimensional Coulomb gas, and the interfacial roughening transition. Phys. Rev. B.

[6] Knops HJF (1977). Exact Relation between the Solid-on-Solid Model and the XY Model. Phys. Rev. Lett..

[7] van Beijeren H (1977). Exactly solvable model for the roughening transition of a crystal surface. Phys. Rev. Lett..

[8] Weeks, J. D. Ordering in Strongly Fluctuation Condensed Matter Systems. (ed. Riste, T.) 293–317 (Plenum: New York, London, 1980).

[9] Huse DA, van Saarloos W, Weeks JD (1985). Interface Hamiltonians and bulk critical behavior. Phys. Rev. B.

[10] Barabasi AL, Stanley HE (1995). Fractal Concepts in Surface Growth.

[11] Krug J, Spohn H, Godrèche E (1991). Kinetic roughening of growing surfaces. Solids Far From Equilibrium.

[12] Takeuchi KA (2013). Crossover from growing to stationary interfaces in the Kardar–Parisi–Zhang Class. Phys. Rev. Lett..

[13] Takeuchi KA (2018). An appetizer to modern developments on the Kardar-Parisi-Zhang universality class. Physica A.

[14] Kardar M, Parisi G, Zhang Y-C (1986). Dynamic Scaling of Growing Interfaces. Phys. Rev. Lett..

[15] Pagnani A, Parisi G (2015). Numerical estimate of the Karder-Parisi-Zhang universality class in (2+1) dimensions. Phys. Rev. Lett..

[16] Krim J, Palasantzas G (1995). Experimental observations of self-affine scaling and kinetic roughening at sub-micron length scales. Int. J. Mod. Phys. B.

[17] Villain J (1991). Continuum models of crystal growth from atomic beams with and without desorption. J. Phys..

[18] Pimpinelli A, Villain J (1998). Physics of Crystal Growth.

[19] Einstein TL, Pierre-Louis O (1999). Implications of random-matrix theory for therrace-width distributions on vicinal surfaces: improved approximations and exact results. Surf. Sci. Lett..

[20] Pimpinelli A, Tonchev V, Videcoq A, Vladimirova M (2002). Scaling and Universality of self-organized patterns on unstable vicinal surfaces. Phys. Rev. Lett..

[21] Xia H, Tang G, Lan Y (2020). Long-Range Temporal Correlations in Kinetic Roughening. J. Stat. Phys..

[22] dn Nijs M, Riedel EK, Conrad EH, Engel T (1985). Roughening of stepped metal surfaces. Phys. Rev. Lett..

[23] Akutsu N, Akutsu Y (1987). Roughening, faceting and equilibrium shape of two-dimensional anisotropic interface. I. Thermodynamics of interface fluctuations and geometry of equilibrium crystal shape. J. Phys. Soc. Jpn..

[24] Yamamoto T, Akutsu Y, Akutsu N (1994). Fluctuation of a Single Step on the Vicinal Surface -Universal and Non-Universal Behaviors. J. Phys. Soc. Jpn..

[25] Akutsu Y, Akutsu N, Yamamoto T (1994). Logarithmic step fluctuations in vicinal surface: a Monte Carlo study. J. Phys. Soc. Jpn..

[26] Wolf DE, Villain J (1990). Growth with surface diffusion. Europhys. Lett..

[27] Sarma SD, Tamborenea P (1991). A New Universality class for kinetic growth: one-dimensional Mcular-Beam Epitaxy. Phys. Rev. Lett..

[28] Lai Z-W, Sarma SD (1991). Kinetic growth with surface relaxation: continuum versus atomistic models. Phys. Rev. Lett..

[29] Sarma SD, Ghaisas SV (1992). Solid-on-solid rules and models for nonequilibrium growth in 2+1 dimensions. Phys. Rev. Lett..

[30] Plischke, M., Shore, J. D., Schroeder, M., Siegert, M. & Wolf, D. E. Comment on “Solid-on-Solid rules and Models for Nonequilibrium Growth in 2+1 Dimensions”. *Phys. Rev. Lett.* **71**, 2509–2509 (1993). 10.1103/PhysRevLett.71.2509 10054698

[31] Amar JG, Lam Pui-Man, Family F (1993). Groove instabilities in surface growth with diffusion. Phys. Rev. E.

[32] Sarma SD, Ghaisas SV, Kim JM (1994). Kinetic super-roughening and anomalous dynamic scaling in nonequilibrium growth models. Phys. Ref. E.

[33] Smilauer P, Kotrla M (1994). Crossover effects in the Wolf-Villain model of epitaxial growth in 1;1 and 2+1 dimensions. Phys. Rev. B.

[34] Krasteva A, Popova H, Akutsu N, Tonchev V (2016). Time scaling relations for step bunches from models with step-step attractions (B1-type models). AIP Conf. Proc..

[35] Gupta I, Mohanty BC (2016). Dynamics of surface evolusion in semiconductor thin films grown from a chemical bath. Sci. Rep..

[36] Mitani T, Komatsu N, Takahashi T, Kato T, Harada S, Ujihara T, Matsumoto Y, Kurashige K, Okumura H (2015). Effect of aluminum addition on the surface step morphology of 4H-SiC grown from Si-Cr-C solution. J. Cryst. Growth.

[37] Akutsu N (2009). Thermal step bunching on the restricted solid-on-solid model with point contact inter-step attractions. Appl. Surf. Sci..

[38] Akutsu N (2011). Non-universal equilibrium crystal shape results from sticky steps. J. Phys. Condens. Matter.

[39] Akutsu N, Yamamoto T, Nishinaga T (2015). Rough-Smooth Transition of Step and Surface. Handbook of Crystal Growth.

[40] Akutsu N (2016). Faceting diagram for sticky steps. AIP Adv..

[41] Akutsu N (2017). Profile of a faceted macrostep caused by anomalous surface tension. Adv. Condens. Matter Phys..

[42] Akutsu N (2017). Disassembly of faceted macrosteps in the step droplet zone in non-equilibrium steady state. Crystals.

[43] Akutsu N (2018). Height of a faceted macrostep for sticky steps in a step-faceting zone. Phys. Rev. Mater..

[44] Akutsu N (2019). Relationship between macrostep height and surface velocity for a reaction-limited crystal growth process. Cryst. Growth Des..

[45] Akutsu N (2019). Driving force dependence of the height of a faceted macrostep in nonequilibrium steady-state crystal growth. J. Phys. Conf. Ser..

[46] Chernov AA (1961). The spiral growth of crystals. Sov. Phys. USP.

[47] Sogo K, Akutsu Y, Abe T (1983). New factorized S-matrix and its application to exactly solvable q-state model. II. Prog. Theor. Phys..

[48] den Nijs M (1985). Exact solubility of the self-dual and the string melting points in the restricted solid-on-solid model. J. Phys. A. Math. Gen..

[49] Akutsu Y (1989). Exact Landau free-energy of solvable N-state vertex model. J. Phys. Soc. Jpn..

[50] Kim JM, Kosterlitz JM (1989). Growth in a restricted solid-on-solid model. Phys. Rev. Lett..

[51] Müller-Krumbhaar H, Kaldis E (1978). Kinetics of crystal growth. Current Topics in Materials Science.

[52] Wolf DE (1991). Kinetic Roughening of Vicinal Surface. Phys. Rev. Lett..

[53] Krzyzewski F, Zaluska-Kotur MA (2014). Coexistence of bunching and meandering instability in simulated growth of 4H-SiC(0001) surface. J. Appl. Phys..

[54] MacKenzie JK, Moore AJW, Nicholas JF (1962). Bonds broken at atomically flat crystal surfaces-I: face-centred and body-centred cubic crystals. J. Chem. Phys. Solids.

[55] Akutsu N, Akutsu Y (1999). Statistical mechanical calculation of anisotropic step stiffness of a two-dimensional hexagonal lattice-gas model with next-nearest-neighbor interactkions: application to Si(111) surface. J. Phys. Condens. Matter.

[56] Akutsu N (2014). Measurement of microscopic coupling constants between atoms on a surface: combination of LEEM observation with lattice model analysis. Surf. Sci..

[57] Ehrlich G, Hudda FG (1966). Atomic view of surface self-diffusion: Tungsten on Tungsten. J. Chem. Phys..

[58] Schwoebel RL, Shipsey EJ (1966). Step motion on crystal surfaces. J. Appl. Phys..

[59] Alerhand OL, Vanderbilt D, Meade RD, Joannopoulos JD (1988). Spontaneous formation of stress domains on crystal surfaces. Phys. Rev. Lett..

[60] Williams, E. D., Phaneuf, R. J., Wei, J., Bartelt, N. C. & Einstein, T. L. Thermodynamics and statistical mechanics of the faceting of stepped Si (111). *Surf. Sci.* **294**, 219–242 (1993). Erratum to “Thermodynamics and statistical mechanics of the faceting of stepped Si (111)” [ Surf. Sci. 1993, 294, 219], Surf. Sci. **310**, 451 (1994).

[61] Akutsu N, Akutsu Y, Yamamoto T (2001). Stiffening transition in vicinal surfaces with adsorption. Prog. Theory Phys..

[62] Akutsu N, Akutsu Y, Yamamoto T (2001). Vicinal surface with Langmuir adsorption: a decorated restricted solid-on-soli model. Phys. Rev. B.

[63] Akutsu N, Akutsu Y, Yamamoto T (2003). Thermal step bunching and interstep attraction on the vicinal surface with adsorption. Phys. Rev. B.

[64] Akutsu N, Hibino H, Yamamoto T (2009). A lattice model for thermal decoration and step bunching in vicinal surface with sub-monolayer adsorbates. e-J. Surf. Sci. Nanotechnol.

[65] Kempisty P, Kangawa Y (2019). Evolution of the free energy of the GaN(0001) surface based on first-principles phonon calculations. Phys. Rev. B.

[66] Kreyszig E (1968). Introduction to differential geometry and riemannian geometry.

[67] Akutsu N, Sugioka Y, Murata N (2020). Surface roughness changes induced by stoichiometric deviation in ambient phase for two-component Semiconductor Crystals. Crystals.

[68] Akutsu Y, Akutsu N, Yamamoto T (1988). Universal jump of Gaussian curvature at the facet edge of a crystal. Phys. Rev. Lett..

[69] Yamamoto T, Akutsu Y, Akutsu N (1988). Universal behavior of the equilibrium crystal shape near the facet edge. I. A generalized terrace-step-Kink Model. J. Phys. Soc. Jpn..

[70] Zhang H, Wang H, Kuang W, Zhang Y, Li S, Zhao Y, Herlach DM (2018). Rapid solidification of non-stoichiometric intermetallic compounds: modeling and experimental verification. Acta Materialia.

[71] Gilmer GH, Bennema P (1972). Simulation of crystal growth with surface diffusion. J. Appl. Phys..

[72] Müller-Krumbhaar H, Binder K (1979). Monte Carlo Simulation of Crystal Growth. Monte Carlo Methods in Statistical Mechanics.

[73] Ohta T, Jasnow D, Kawasaki K (1982). Universal scaling in the motion of random interfaces. Phys. Rev. Lett..

[74] Gruber EE, Mullins WW (1967). On the theory of anisotropy of crystalline surface tension. J. Phys. Chem. Solids.

[75] Pokrovsky VL, Talapov AL (1979). Ground state, spectrum, and phase diagram of two-dimensional incommensurate crystals. Phys. Rev. Lett..

[76] Jayaprakash C, Rottman C, Saam WF (1984). Simple model for crystal shapes: step-step interactions and facet edges. Phys. Rev. B.

[77] den Nijs M, Rommelse K (1989). Preroughening transitions in crystal surfaces and valence-bond phases in quantum spin chains. Phys. Rev. B.

