# Supplementary material for: Crossover from BKT-rough to KPZ-rough surfaces for interface-limited crystal growth/recession
Source: Sci Rep. 2020 Aug 3;10:13057. doi: 10.1038/s41598-020-70008-y (PMC7400654; doi:10.1038/s41598-020-70008-y)
Supplement: Supplementary file 1 — Supplementary information. [file 41598_2020_70008_MOESM1_ESM.pdf]

Crossover from BKT-Rough to KPZ-Rough Surfaces for Interface-Limited Crystal Growth/Recession

[1*)]Noriko Akutsu
[1)]*Faculty of Engineering, Osaka Electro-Communication University, Hatsu-cho, Neyagawa, Osaka 572-8530, Japan*

[1*] nori3@phys.osakac.ac.jp

S1 Model

The model that the Monte Carlo treatment in this study uses is based on the RSOS model on a square lattice. Here, "restricted" means that the surface height difference between nearest neighbor sites is restricted to 0 or ±1. The surface Hamiltonian for the RSOS model is given by the following equation:

$$\mathcal{H} = \sum_{\{m,n\}} \{\epsilon[|h(m+1,n) - h(m,n)| + |h(m,n+1) - h(m,n)|] - \Delta\mu\ h(m,n)\} + \mathcal{N}E_{\rm surf}, \qquad (1)$$

where $h(m,n)$ is the height of the surface at a site (n,m), ϵ is the microscopic ledge energy, $\mathcal{N}$ is the total number of the unit cells on the (001) surface, and $E_{\rm surf}$ is the surface energy per unit cell. The RSOS condition is required implicitly. Here, $\Delta\mu$ is introduced such that $\Delta\mu = \mu_{\rm ambient} - \mu_{\rm crys}$, where $\mu_{\rm ambient}$ and $\mu_{\rm crys}$ are the bulk chemical potential of the ambient and crystal phases, respectively. At equilibrium, $\Delta\mu = 0$; for $\Delta\mu > 0$, the crystal grows; whereas $\Delta\mu < 0$, the crystal recedes.

For the first-principles quantum mechanical calculations, $E_{\rm surf}$ corresponds to the surface free energy, which includes entropy originating from lattice vibrations and distortions. Hence, $E_{\rm surf}$ or ϵ slightly decreases as the temperature increases. However, $E_{\rm surf}$ and ϵ are assumed to be constant throughout the work because we concentrate on the crossover phenomena of the surface roughness.

S2 Monte Carlo method

Vicinal surfaces of a (001) surface tilted towards the [111] direction are considered using the Monte Carlo method for non-conserved systems with the Metropolis algorithm. Here, "non-conserved" indicates that the number of crystals is not conserved. Atoms on the crystal surface can escape to the ambient phase or atoms in the ambient phase can be captured. The external parameters are temperature T, $\Delta\mu$, number of steps $N_{\rm step}$, and the linear size of the system L. The surface slope $p = N_{\rm step}a/L = \tan\theta$, where θ is the tilt angle from the $\langle 001\rangle$ direction.

Initially, $N_{\rm step}$ steps run in the mean direction $\tilde{y} = \langle\bar{1}10\rangle$, for which a periodic boundary condition is required. The $\tilde{x}$ direction is assigned to the $\langle 110\rangle$ direction. The configurations on the lower height side (right side of the top-down view of the surface) are connected to the upper height side (left side of the top-down view of the surface) by adding $N_{\rm step}a$ steps. We considered two types of initial surface configuration: a surface with a macrostep where all the steps are combined , and a surface with a train of steps. After 2×10^8 Monte Carlo steps per site (MCS/site), the results from both initial conditions agree well.

The lattice sites on the surface at which an event occurs are selected randomly and a "capture" or "escape" event for the lattice site is selected randomly with a probability of 1/2. The energy change between the states before and after an event is calculated using Eq. (1). If the energy change is negative, the surface configuration is updated with a probability of 1. If the energy change is positive, the surface configuration is updated with a probability of $\exp[-(E_{\rm after} - E_{\rm before})/k_{\rm B}T]$. Here, $E_{\rm after}$ and $E_{\rm before}$ are the surface energies calculated using Eq. (1) after and before the surface configuration update, respectively.

The surface diffusion of atoms or volume diffusion are not taken into consideration. The advance and recession of an elementary step are respectively caused by the capture and escape of atoms at kinks on the step edges. In the following, snapshots of the top-down and side views of the simulated surfaces are shown.

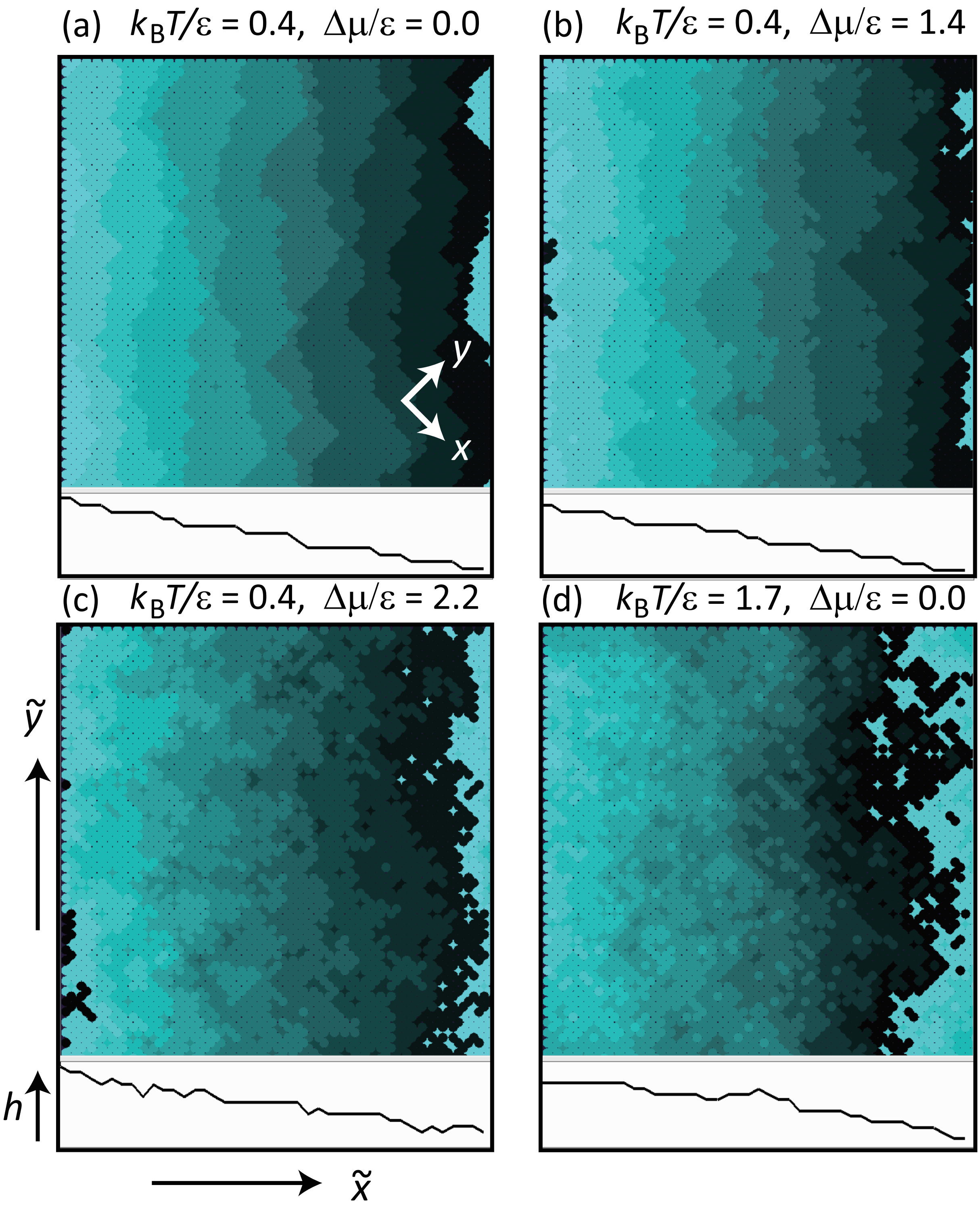

Figure S1. Snapshot of simulated surfaces at 4×10^8 MCS/site. Size: $40\sqrt{2} \times 40\sqrt{2}$. N_{step}=10. $p = N_{\mathrm{step}}a/L = \sqrt{2}/8 \approx 0.177$. $\theta = 10.0$ degree. The surface height is represented by brightness with 10 gradations, where brighter regions are higher. Due to the finite gradation, where the darkest areas sit next to the brightest areas, the darker area is higher by one gradation unit. The lines of the side view are drawn with respect to the height along the bottom edge of the top-down view. All the surfaces are BKT-rough.

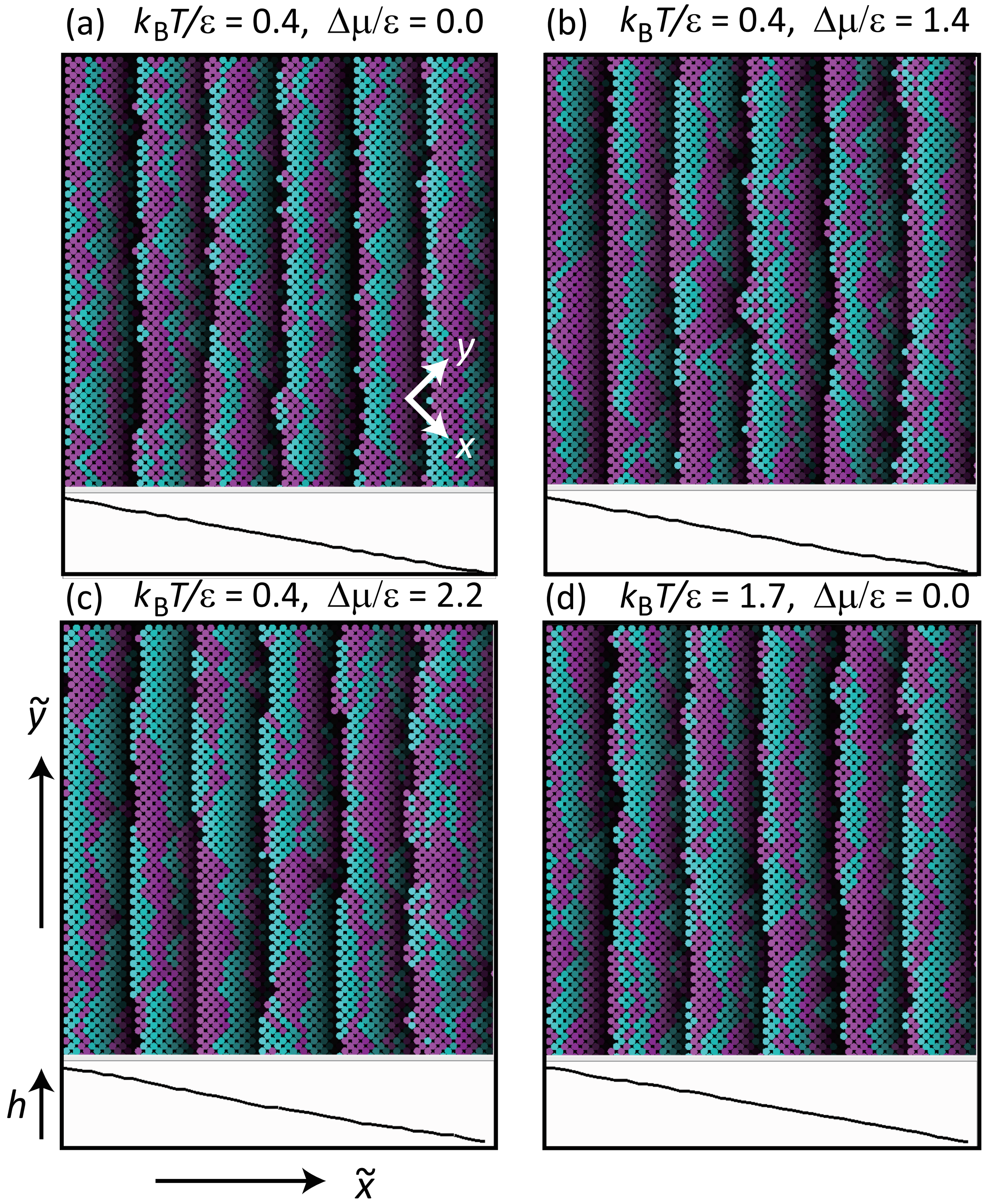

Figure S2. Snapshot of simulated surfaces at 4×10^8 MCS/site. Size: $40\sqrt{2} \times 40\sqrt{2}$. $N_{\rm step}$=60. $p = 3\sqrt{2}/4 \approx 1.061$. $\theta = 46.7$ degree. The (111) terrace-layers are colored blue or red, alternatively. (a) and (d) are BKT-rough, (b) and (c) are KPZ-rough.

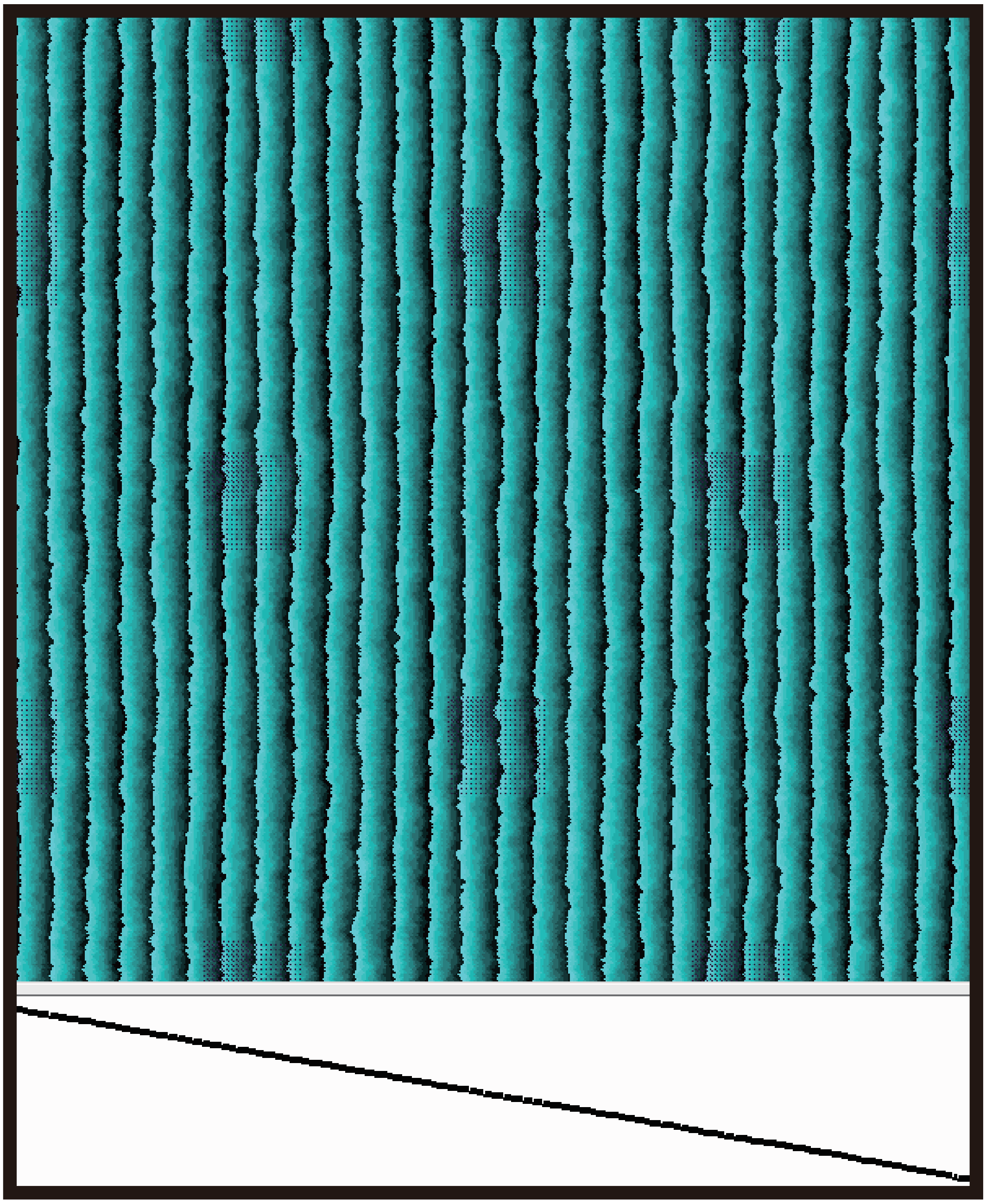

Figure S3. Snapshot of a simulated surface at 4×10^8 MCS/site. $k_{\rm B}T/\epsilon = 0.4$. $\Delta\mu = 0$. Size: $400\sqrt{2} \times 400\sqrt{2}$. $N_{\rm step}$=300. $p = 3\sqrt{2}/8 \approx 0.530$. $\theta = 27.9$ degree. The surface is BKT-rough.

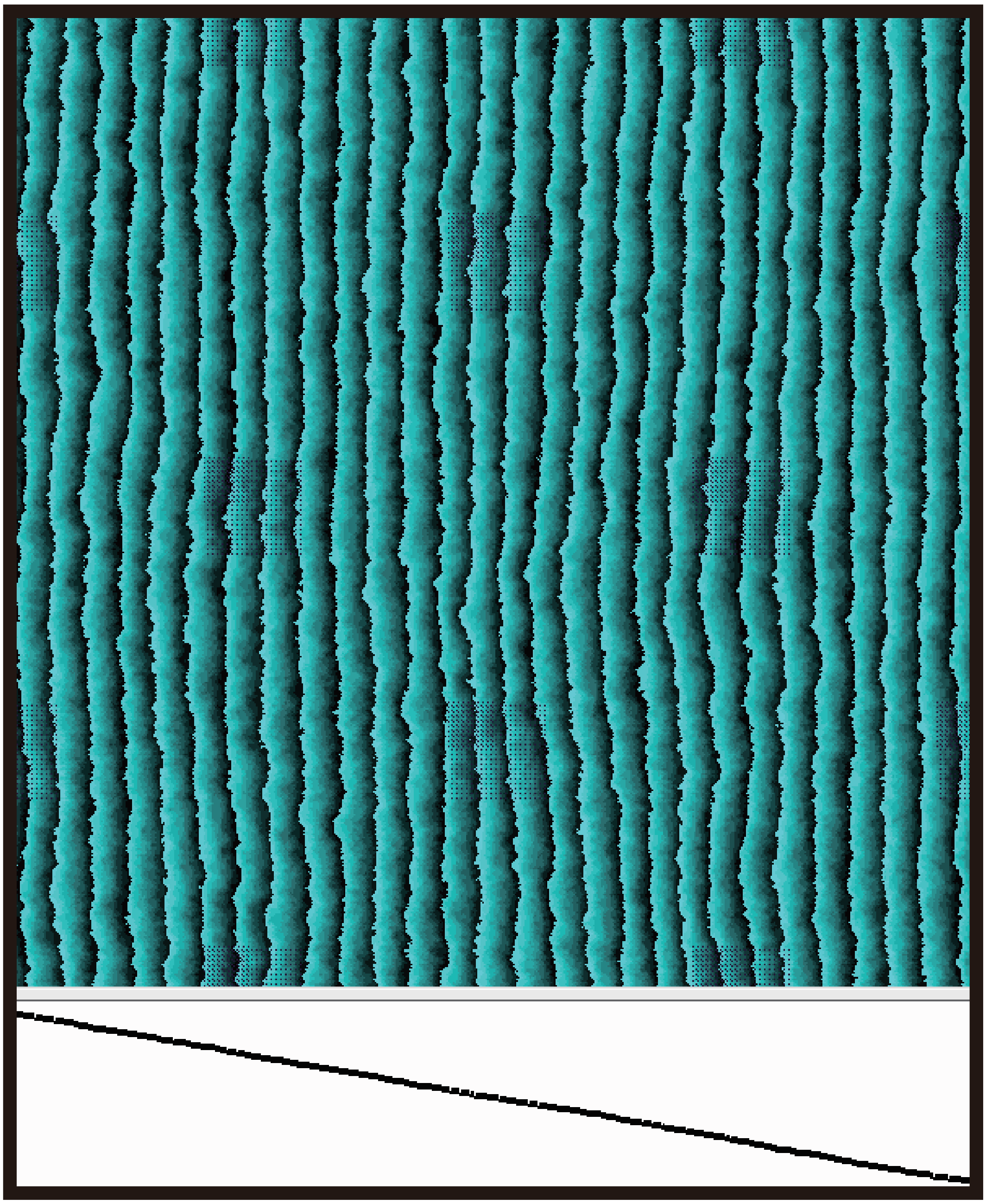

Figure S4. Snapshot of a simulated surface at 4×10^8 MCS/site. $k_{\rm B}T/\epsilon = 0.4$. $\Delta\mu/\epsilon = 1.4$. Size: $400\sqrt{2} \times 400\sqrt{2}$. $N_{\rm step}$=300. $p = 3\sqrt{2}/8 \approx 0.530$. $\theta = 27.9$ degree. The surface is KPZ-rough.

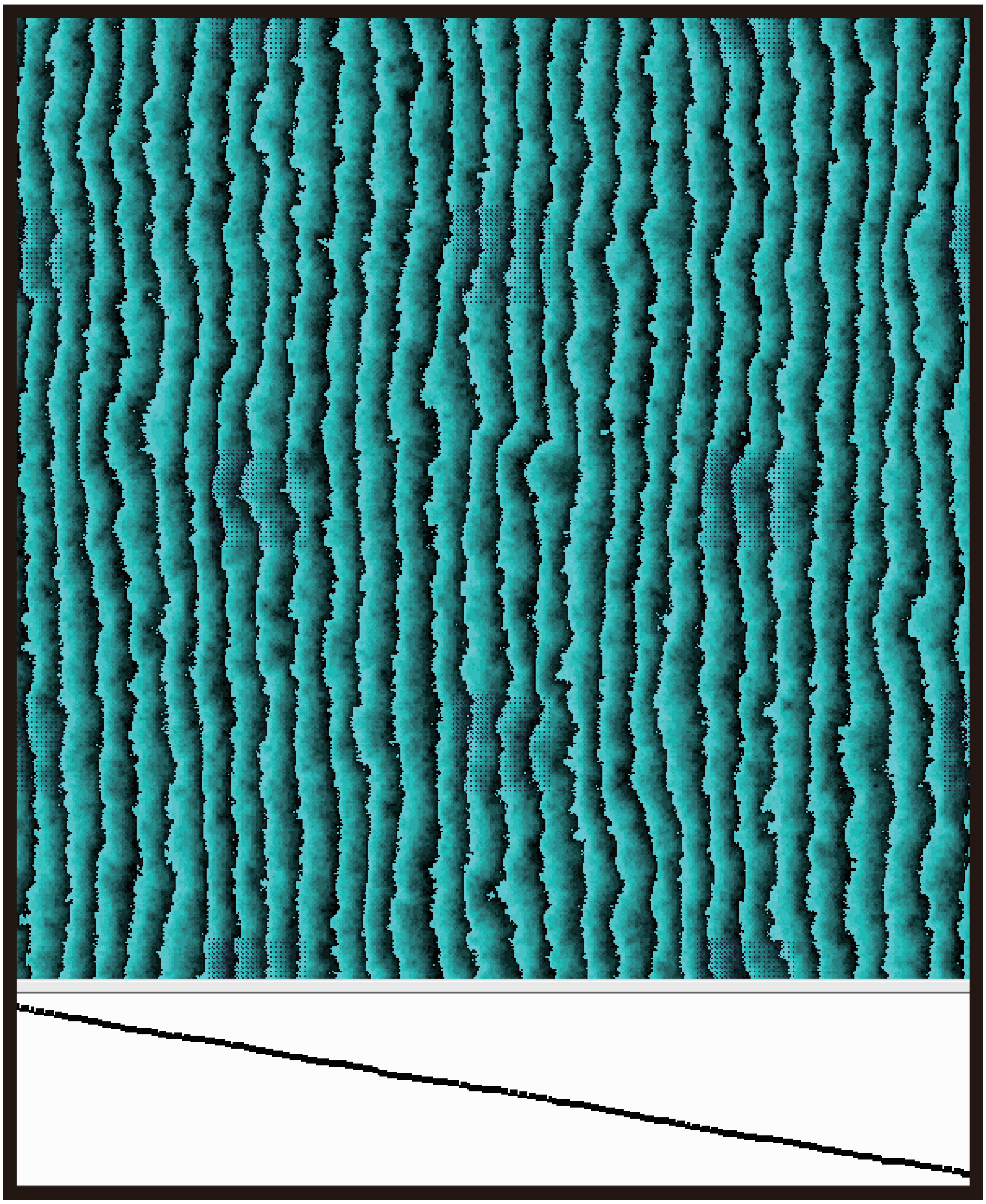

Figure S 5. Snapshot of a simulated surface at 4×10^8 MCS/site. $k_{\rm B}T/\epsilon = 0.4$. $\Delta\mu = 2.2$. Size: $400\sqrt{2} \times 400\sqrt{2}$. $N_{\rm step}$=300. $p = 3\sqrt{2}/8 \approx 0.530$. $\theta = 27.9$ degree. The surface is KPZ-rough.

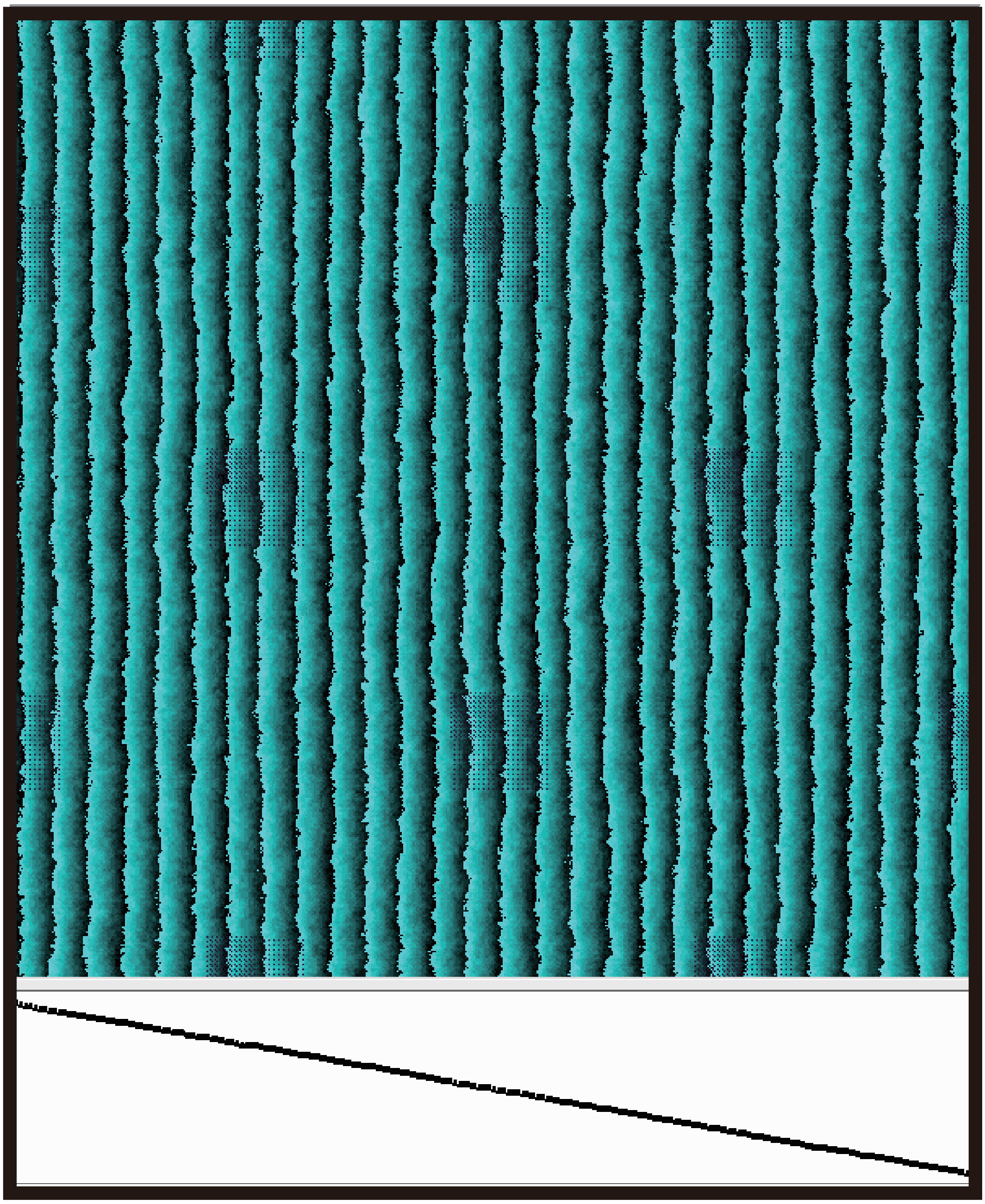

Figure S 6. Snapshot of a simulated surface at 4×10^8 MCS/site. $k_{\rm B}T/\epsilon = 1.7$. $\Delta\mu = 0.03$. Size: $400\sqrt{2} \times 400\sqrt{2}$. $N_{\rm step}$=300. $p = 3\sqrt{2}/8 \approx 0.530$. $\theta = 27.9$ degree. The surface is BKT-rough.
